# Cascade of flight interception traps for large scale exploration of the otherwise unreachable canopy insect fauna

**DOI:** 10.1038/s41598-025-19981-w

**Published:** 2025-10-15

**Authors:** José A. Rafael, Francisco Limeira-de-Oliveira, Ismael B. Oliveira, Sheila P. Lima, Alice Tôrres, Daniell R. R. Fernandes, Dalton S. Amorim

**Affiliations:** 1 National Institute of Amazonian Research, Manaus, Brazil; 2 State University of Maranhão, Caxias, Brazil; 3 Entomology Grad Program, Department of Biology, FFCLRP, University of São Paulo, Ribeirão Preto, Brazil

**Keywords:** Zoology, Entomology

## Abstract

We present here an efficient system of flight intercept traps developed to be set at the forest structure in tropical or temperate environments to assess the vertical structure of the insect fauna, including the ground level and the canopy at different heights. The system does not demand a tower or a crane to sustain the traps, and there is no need for a climber to fix the system at a higher branch in the canopy. The trap is a modified model of the Gressitt and Gressitt flight interception trap especially adjusted to a hanging set of traps here called the cascade system. Trap integration is a novel solution for exploring the canopy fauna. Initial counts of insect numbers at various heights, from ground level to the canopy, showed the efficiency of the cascade system in all strata explored. Finding means to access the unreachable fauna of the canopy is necessary, and the cascade system can be replicated worldwide in any forest to explore insect diversity and its dynamics.

**Supplementary Information:**

The online version contains supplementary material available at 10.1038/s41598-025-19981-w.

## Introduction

Beebe^1^ long ago clearly stated that assessing the insect canopy fauna was like discovering the fauna of a new continent—i.e., an exquisite, particularly distinct fauna. Over one century later, we are still much at the same place. Logistics to sample the canopy insect fauna is particularly complex, and our understanding of the dynamics and complexity of this fauna is still limited. Ever since Beebe’s^1^ comments, there have been efforts to access the forest canopy. Sweeping directly at the canopy using a portable rope ladder^2^, suspended platform^3^, chemical knockdowns (or fogging)^4^, and suspended traps^5,6^ have been used, but there are still no particularly efficient methods designed for continuous extensive scale assessment of the canopy insect fauna at different heights of the forest.

Erwin^4^, over 40 years ago, predicted that there may be as many as 30 million species of insects in the world, using as basis for his calculations the number of forest types and assumptions of associations between phytophagous insects and tree species. Erwin^4,7^ used fogging to collect insects in the forest, which is efficient but loses the vertical stratification signal of the fauna.

Most projects addressing canopy insect fauna have a taxonomic focus—drosophilids^8^, hymenopterans^9^, heteropterans^10^, bees^11^, beetles and flies^12^, and moths^13^. However, some extensive inventories have detailed results only for taxa with specialists available [e.g., ^14,15^]. Indeed, dealing with the complete samples of the insect canopy fauna corresponds itself to a separate issue. It is necessary the joint effort of a large network of specialists to give meaning to canopy samples, with thousands of species of hundreds of insect families, while morphology alone makes the process too slow, costly, and time-consuming. Additional solutions to fulfill this part of the challenge are necessary.

The question of having target taxa in canopy studies naturally brought up technical sampling solutions, and traps for certain groups of insects are well developed. There is still a technical gap, however, for large-scale sampling of the insect canopy fauna keeping the stratification information. The point of departure for solutions to large-scale insect sampling is René Malaise’s flight intercept trap, who developed the concept and the first models^16^—these traps now bearing his name. This trap is a stationary, tent-like lightweight trap open on both sides, with a central panel and a top-mounted collecting apparatus. It is a non-attractant device, based upon the principle that most phototropic-positive flying insects hitting an obstacle respond by flying (or crawling) upwards, ending up in the collection apparatus above^17^. The trap material color blends with the background and allows the free flow of air through the fine mesh.

Several modifications have been proposed over the years to improve the efficiency of the traps^18–25^. Eventually, these models are referred to by the name of the author(s) that developed such modifications, such as the Townes or the Gressitt and Gressitt trap models. The first models were built to explore insects flying near the ground stratum, sometimes set a few meters above the understory level. Skvarla et al.^26^ and Uhler et al.^27^ presented detailed revisionary papers comparing the different interception trap variants.

As our understanding of the uniqueness of the canopy insect fauna advanced, it was necessary to adapt these traps to explore the canopy^28^. Adaptations of flight intercept traps were always made following the principle that specimens generally climb up after encountering a barrier and drop in the collection jar or apparatus. These successive changes in the original model are a testament to the versatility and effectiveness of the interception flight trap in various ecological niches.

One of the first collections using modified flight interception traps to collect in the canopy was made by Crossley et al.^5^, who constructed platforms in the middle of the canopies. The first “free” suspended Malaise trap to collect high in the forest, sustained by ropes, was designed by Rafael and Gorayeb^6^. They developed a simple model lifted to sample mostly tabanids and some other groups of insects in the canopy. This involved the construction of a lightweight but sturdy trap that ropes could suspend. That model later received adjustments and modifications^29^, and commercial models can now be acquired and placed high in the forest.

Our experience collecting in the Amazon over the years showed that the most efficient and easily mounted reference trap for large-scale sampling at the canopy is the Gressitt and Gressitt^20^ large-size (5–6 m long, 3–3.5 m high) model, with dual collection heads. It is a tent-like understory trap that can be mounted using ropes over tree branches of appropriate height and tied to low branches or tree trunks.

That model was used to document the vertical stratification of the insect fauna across a vertical gradient in a Central Amazonian tropical forest^15^: five traps were set at five levels using the platforms (at every 8 m) of a 40-m-high steel tower. The results of a single set of two-week samples collected at five levels of the tower^15^ corroborated previous reports [e.g., ^30,31^] that the Diptera guild structure and taxonomic composition are particularly distinct across the vertical structure of the forest. It is no overstatement that the canopy harbors a distinct set of taxa that can be largely called a fauna of its own. Steel towers (or cranes), however, are uncommon in tropical forests around the world. We need scalable solutions for efficient trapping at the canopy to study the vertical turnover of the insect fauna in different parts of the Amazon. This paper describes the modifications made to the trap and the system to lift them in a verticalized “cascade” using an emergent tree as the base to support the traps. The material we used can be changed according to each group’s necessities. The description below will be helpful in further installation to explore the stratified fauna in the forests.

## Results

This trap model is the result of improvements on a number of previous versions of hanging flight interception traps we worked on. A first assessment of the trap catches at three different collecting sites at different season with the five trap levels show quite impressive numbers (Table 1). The number of specimens collected by our cascade system in 14 days by the set of five trap levels varied from 38,000 to over 82,000 specimens, with a mean of almost 59,000 specimens. These numbers are quite surprising even compared to our previous study^15^, in which the metal tower in the ZF2 Biological Reserve was used to fix the traps at similar heights, the set of traps at five levels yielding 37,000 specimens in 2 weeks. The mean catches in 2 weeks at the ground level trap was of 1345 specimens/trap/day, 4210 specimens/trap/day for the entire system. The expected number of specimens sampled is crucial for the design of large-scale tropical forest inventories as it impacts the estimate of the overall project costs, including time to process samples, sorting and/or sequencing, and the need for subsampling.

Comparisons between the mean abundance of the samples of the five forest levels in our trap system indicate that all four higher levels individually collect less than the ground level sample. However, the ground level samples represent less than one-third of the overall number of specimens collected above the ground level (Table 1). In terms of species richness, our previous study in the ZF2 reserve in Manaus^15^ shows that about 62% of the fly species were not sampled at the ground level trap and we now will be able to check how different the canopy fauna is for all insect groups using a COI molecular approach, with a network of specialists identifying the genus of each cluster, in a very embracing approach of the forest overall insect fauna.

**Table 1:** Trap efficiency measured by the number of specimens collected by the traps using the cascade system in the Amazon forest in three collecting points, ZF2 (north of Manaus) and Iranduba (west of Rio Negro) (both in the state of Amazonas) and Gurupi National park (state of Maranhão), Brazil.

Height (meters)	Number of specimens sampled each 15 days											
	ZF2^1^	ZF2^2^	ZF2^3^	ZF2^4^	ZF2^5^	ZF2^6^	Iranduba^7^	Iranduba^8^	Gurupi^9^	Mean	% of level	Specimens/day
Ground level	23,180	14,060	25,460	23,180	15,580	20,900	15,892	18,620	12,626	18,833	**32.0%**	1345
7	15,960	11,400	12,920	8360	7220	7220	13,200	15,960	8404	11,183	**19.0%**	799
14	16,720	8360	4180	3800	4560	7980	10,653	12,540	9410	8689	**14.7%**	621
21	1520^10^	22,420	17,860	7600	7220	4180	8716	17,480	8463	10,607	**18.0%**	758
28	14,440	14,820	6460	4180	3420	6840	9396	17,480	9664	9633	**16.3%**	688
**Total**	**71,820**	**71,060**	**66,880**	**47,120**	**38,000**	**47,120**	**57,857**	**82,080**	**48,567**	**58,945**	–	**4210**

Collecting dates: ^1^23Jul2024; ^2^06Aug2024; ^3^17Sep2024; ^4^12Nov2024; ^5^7Jan2025; ^6^4Mar2025; ^7^22Jul2024; ^8^16Sep2024; ^9^15Feb2024; ^10^trap damaged.

## Discussion

Despite the almost universal use of flight interception traps in insect faunal surveys and that it is well known that there is a vertical turnover of the insect fauna towards the canopy in forests, access itself to the canopy has had limited progress in over a century. Our cascade system of traps allows sampling in widely different environments, a relatively cheap solution compared to building a tower or the use of a crane. This solution for canopy sampling will be valuable in long-term studies of insect faunal composition, seasonality studies, species diversity analyses, and ecologically-oriented investigations and biomonitoring.

Our canopy trap system provides a new solution to explore the canopy insect diversity efficiently. The traps keep the principle of passive, non-attractant sampling devices. We assume that the trap floor improves the catches of beetles, but this feature still needs formal testing. Additional improvements can be easily added with baiting etc. The model is slightly more expensive than regular traps because of the modifications needed in each trap and because of the system itself to support the set of traps. It is still light (12 kg for each trap plus the aluminum tubes) and two people can install and process the entire system. In over 6 months of trapping (checked every 2 weeks) the cascade system showed to be suitable in most weather conditions. Exceptions are violent storms, which may partially damage the traps. This happened twice but the samples were not lost, showing resilience of the system.

The first quantitative results make clear the efficiency of the traps in terms of sampled abundance. Further ahead in the development of our project, we will have detailed data also for species richness at the canopy for different insect groups. Provisional data shows that five traps set vertically are much more efficient in terms of sampling forest species-richness than five traps collected at the ground level (personal observations based on groups as tabanids, tachinids, phorids, and mycetophilids). The data for the cascade trap at the ZF2 Biological Reserve (Table 1) so far consists of six samples collected during the dry season (June to November) and three samples during the rainy season (December to May): abundance clearly decreases during the dry season and increases during the rainy season. As mentioned before, the remarkable canopy insect fauna has been referred to as “the unknown world” is referred here as the “unreachable fauna”.

Compared to other generally smaller models of interception flight traps at the ground level, our system is largely more efficient. Skvarla et al.^26^ compiled the number of catches of different studies, but the two larger numbers may have problems. Skvarla et al.’s^26^ reference to Cooksey and Barton’s^32^ results would be of 902 specimens/trap/day over 12 days of sampling—but the actual sampling period was 12 weeks^32^. The study by Brown^33^ would point to 929 specimens/trap/day, but we could not find this number in Brown’s original paper^33^. Excluding the data from these two studies, the largest number reported in Skvarla et al.^26^ based on more than 30 papers was 408 specimens/trap/day. The mean number of specimens collected by our ground level trap is 1345 specimens/trap/day and the overall catches by the system in all five levels is 4210 specimens/trap/day.

The cascade system now allows us to address the insect canopy taxonomic composition to compare different biomes or different areas of endemism. The cascade system actually has to be adapted to specific canopy characteristics, such as canopy height, number of traps and spacing between them along the vertical axis. Forests in different biomes also have stratified environmental conditions and we strongly recommend that each trap level has a climate data logger. In the long run, this will bring up a much better understanding of environmental factors that may affect the insect composition and abundance of different groups at different forest strata. This would also document in the long run how global climate changes impact the microenvironments in the forest.

A couple of ongoing projects financed by the Brazilian government (INCT BioDossel—MCTI/CNPq and BioInsecta—FAPESP) employing these traps should collect along the next 14 months about 7.5 million insects in five forest levels at four sites and sequence the COI barcode of about 600.000 specimens in three Brazilian labs (Instituto Nacional de Pesquisas da Amazônia, Manaus; Universidade de São Paulo, Ribeirão Preto; and Universidade Federal do Rio de Janeiro, Rio de Janeiro). This should bring an unprecedented assessment of tropical taxonomic composition, species richness, taxa abundance, vertical distribution of insect guilds, and vertical and geographical turnover of the insect fauna. This understanding of the fauna is obviously entirely dependent on access to the canopy insect diversity. The system proposed here, hence, pushes forward a new era of insect canopy diversity studies in forests. Such inventory effort is of immediate importance because of the projected rate of species extinctions caused by environmental changes: we urgently need to show politicians and science decision-makers formal numbers of species diversity that could quickly go extinct if we keep habitat degradation. Even mechanisms of forest “protection” that ignore the actual distinctiveness of the canopy fauna could be highly damaging to biodiversity.

## Methods

### Concept

**Fig. 1:**
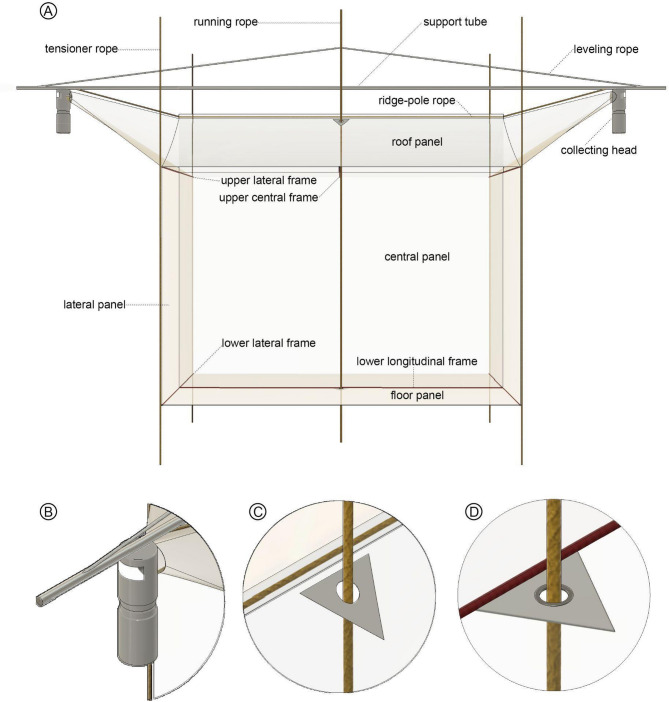
Sketch of the modified Gressitt and Gressitt flight interception trap. (**A**) Trap, showing panels, ropes, tubes and collecting heads. (**B**) Detail of the collecting head. (**C**) Detail of upper triangle hole. (**D**) Detail of lower triangle hole.

Our flight interception trap model to collect insects is primarily based on Gressitt and Gressitt’s [1960] original design. In that paper, they described three models—and our departing point was their type B, which was 5 m long by 3 m high with a cloth floor. It is often made of nylon organdy with two collection heads at the upper lateral ends. It consists of a longitudinal central panel, two transversal lateral panels (Fig. 1A), and sloping roof panels on each side of its top. At each end of the upper corners of the central panel, the joined roof panel and the lateral end extend to form a cone leading to a collection head. The trap is, thus, like a slope-roofed shelter, and the whole net is like a typical ridge-pole house with a central panel instead of front and back walls. It is suspended using stout support and running ropes and opened and stretched using aluminum frame tubes (described below). The trap used in our previous study^15^ was the same model mounted on the tower platforms without stout ropes and without a cloth floor, but in that case, each trap was tied to the tower structure and was set over one of the tower platforms that served as a floor—adding to the catches a considerable number of insects (mainly beetles) that usually evade in regular hanging traps. Sampling the insect fauna at different forest discrete levels demands a set of connected independent traps supported at a higher point and a stabilizing system. The traps and the structure supporting system are described below.

### Net details and collection head adaptations for a stratified vertical system

**Fig. 2:**
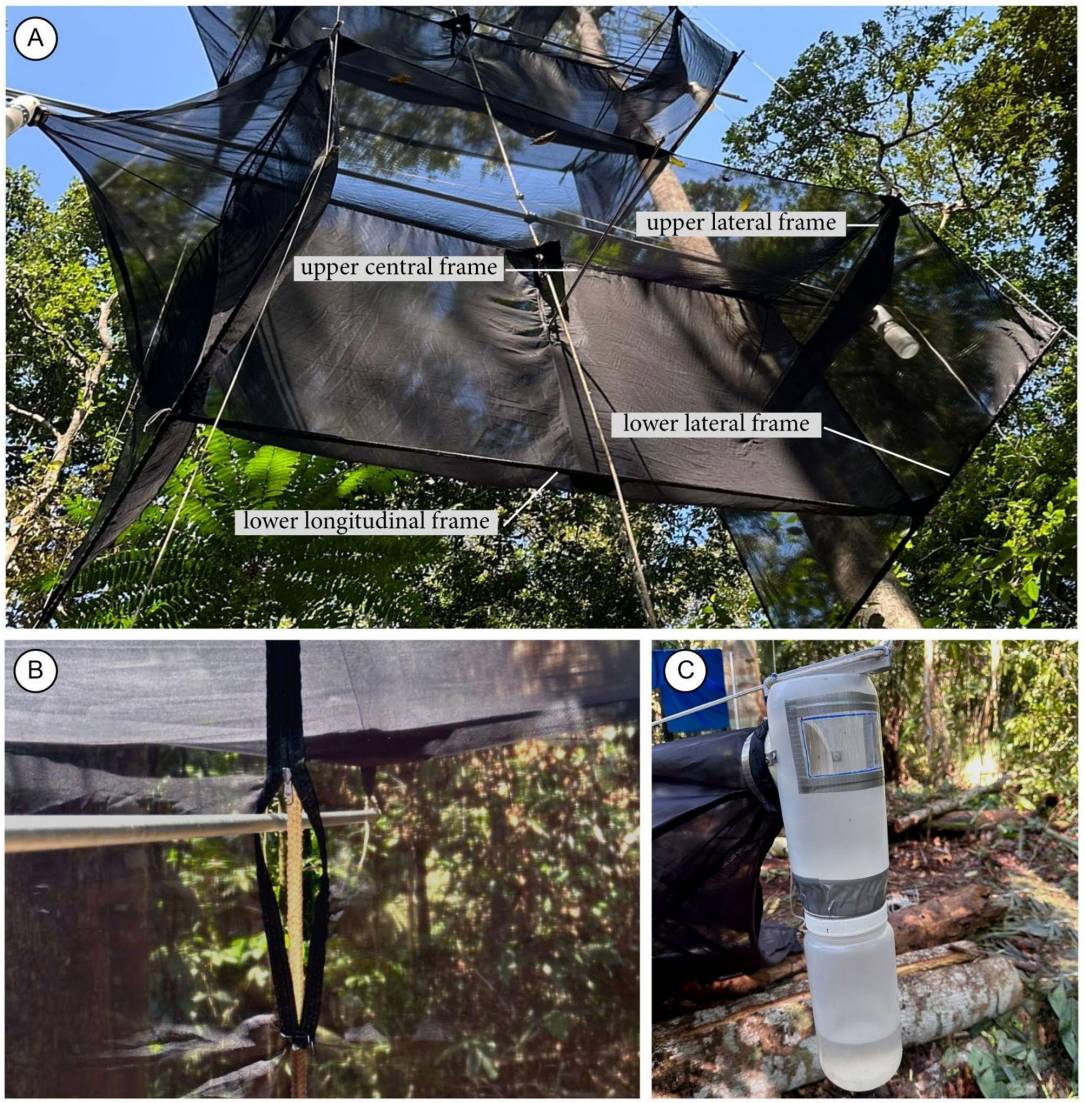
Details of the Gressitt and Gressitt interception flight trap. (**A**) Bottom edge of the trap, showing the aluminum tubes that go through the longitudinal and lateral panels. (**B**) Upper central frame through vertical zipper space. (**C**) Collecting head with detail of the translucent window.

1. Figure 1A shows the names of the trap parts and pieces used along the text. The roof and floor panels have a 40 mm diameter hole on one of the sides, precisely in the middle of the rooftop and on the floor (Fig. 1A–D). The net is reinforced at each hole by a triangular piece (25 cm × 25 cm × 60°) of ripstop fabric, secured between two plastic eyelets.
2. We added 2 cm diameter fabric channels at the bottom edge of the central panel and lateral panels to fit the aluminum tubes, the lower longitudinal, and the lower lateral frames, respectively (Fig. 2A), to keep the trap strained.
3. We added a 60 mm (or less) long vertical zipper (or a velcro fastener) (Fig. 2B) for the upper central frame (Fig. 1A) aluminum tube across the longitudinal central panel, reaching both roof panel lateral edges.
4. Our collection head (Figs. 1A,B and 2C) has a plastic transparent 5 × 13 cm window placed 4 cm below the jar top to increase the light going to the lateral cones at each side. The plastic window is set by gluing a transparent 7 × 16 cm piece of PET (polyethylene terephthalate) over the opening. A 2 × 4.8 mm rivet is fastened at each corner of the plastic piece to fix the transparent window better. A silver tape strip is placed over the junction of the window to avoid water entering the collecting head.
5. We recommend that the longitudinal axis of the trap should be orientated along the east-west axis (sunrise–sunset) in the Amazon basin, with the collection heads receiving the early morning and late afternoon light.

### Trap floor

The floor (already present in the Gressitt and Gressitt’s trap type B) is used in our system (Fig. 1A). It increases the efficiency of the trap, especially for beetles—so its use depends on the questions underlying the project. If the study intends to explore the local insect diversity fully, we highly recommend using trap floors. The floor also contributes to stabilizing and keeping the trap’s shape. The ground trap can be set under the entire system, hanging together with the suspended traps, or can be set apart. In the first case, it allows a more precise comparison of the samples from an ecological point of view. Even at the ground level, the trap floor may bring efficiency.

### Fixing and lifting the traps in a vertical system

**Fig. 3:**
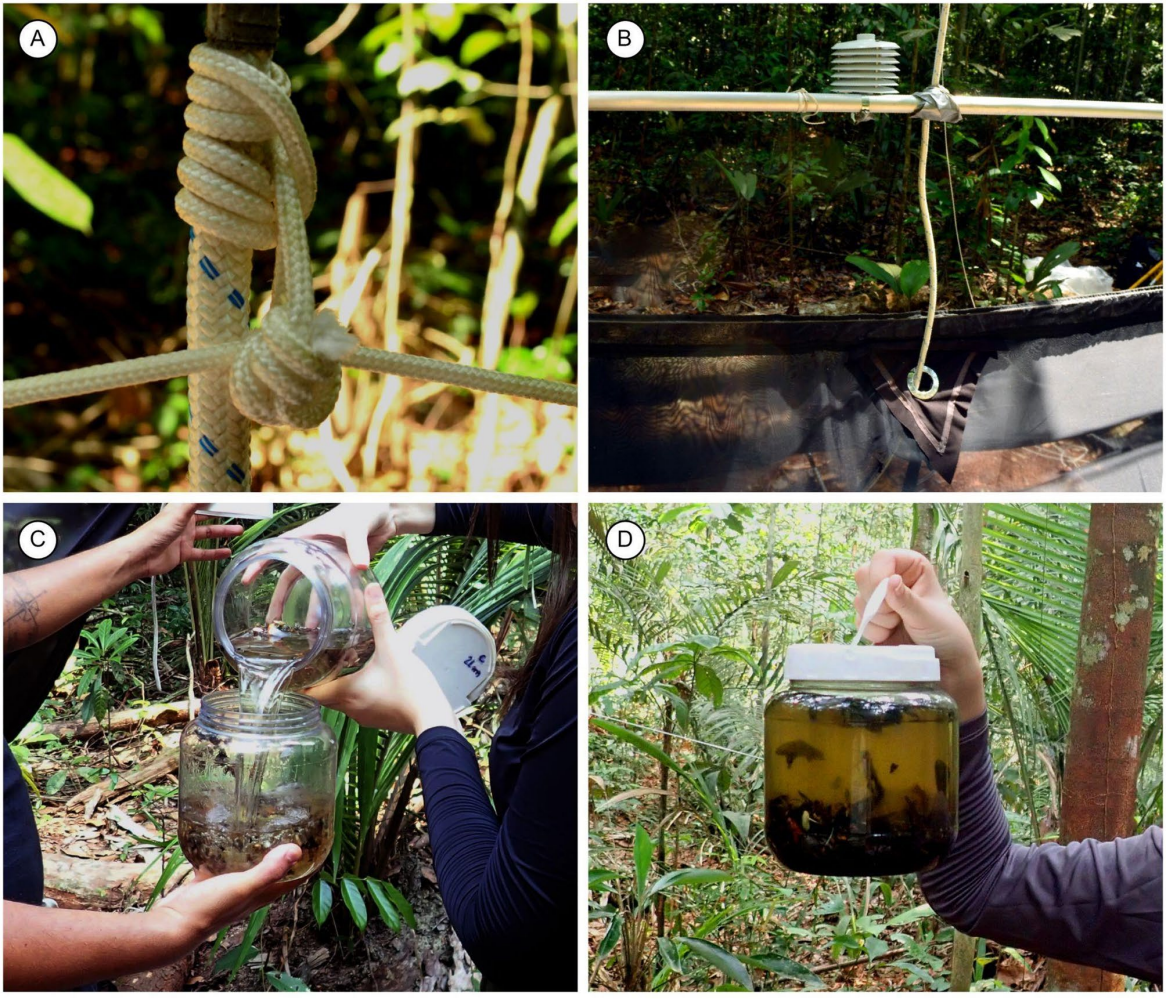
Details of the modified Gressitt and Gressitt interception flight trap. (**A**) Prusik knot. (**B**) Detail of the central upper portion of the trap showing roof hole, data logger on a support tube, and running rope. (**C**) Sample collection. (**D**) Catches with thousands of specimens.

1. We used a support tube (Fig. 1A), a long aluminum tube (6 m long, 30 mm diameter, 3 mm thick wall) to support each trap. The tubes were pierced at 5 cm before both ends to make it easier to tie the ridge-pole rope of the trap. The aluminum tube supports and stabilizes the entire trap.
2. A long, stout, running rope (Fig. 1A) (around double the height of the higher trap, with a polyamide cover, a polypropylene intermediate layer, and a polyamide core) 12 mm diameter, connects all traps of the cascade. It is tied to each trap in the middle of the support tube using a clove hitch knot. After that, lift the support tube one meter high by pulling the other leg of the cascade running rope.
3. Take one trap and fix it to both ends of the support tube by the ridge-pole rope (8 m long, 5 mm diameter). This rope directly supports the trap dorsally (Fig. 1A).
4. Opening the trap. We used five 2-m-long aluminum tubes (10 mm diameter, 1 mm thick wall), with a 5 mm hole (1 cm before each end to tie the lateral loops of the trap) and one 3-m long (same diameter). Three tubes are placed on the roof, with two upper lateral frames at the upper corner of the lateral panel (Fig. 1A) and one upper central frame in the middle across the vertical zipper open space (Figs. 1A and 2B). These tubes are tied to the trap grommets (loops) and keep both sides of the roof panels open and stretched.
5. Pass the running rope through both holes (roof and floor) (Fig. 1A,C,D) of the trap.
6. The support aluminum tube is raised around 2 m high (by pulling the cascade running rope) so the three remaining frame aluminum tubes are threaded into the casing in the bottom edges of the trap. Two of the 2-m-long lower lateral frames are placed at each lower edge of the lateral panel, and one 3-m-long lower longitudinal frame is set across the bottom edge of the central panel (Fig. 1A). All the frame tubes are fixed in the grommets (loops) to stretch the trap.
7. Do a mobile prusik knot and fix it in the cascade running rope (Fig. 3A) just above the attachment point of the cascade running rope with the support aluminum tube. Tie the leveling rope (8 m long, 5 mm diameter) to one end of the support rope, pass the other end through the prusik knot, and tie this end to the other end of the support hope. This leveling rope must be well stretched to keep the support rope on level. When the entire frame is finished, the leveling rope keeps the 6-m tube on level by lifting the mobile prusik knot.
8. Attach the tensioner/stabilizer ropes (four pieces, at least 5 m longer than the height of the traps, 2 mm diameter) at each grommet of the lower corner of the first trap.
9. The first trap is raised high enough by hauling on the cascade running rope to install the next trap, following the steps above. The tensioner ropes are passed through each upper and lower corner grommet (not tied) of each trap (Fig. 1A) to guide it around when it is lifted and lowered and to stabilize it in position in the canopy.
10. If available, fix a data logger climate sensor at the support rope above each trap (Fig. 3B).
11. After all traps are lifted, the cascade running rope loose end and the tensioner/stabilizer cords are tied to reliable, stable anchor points at the ground level.

### Collecting the samples

1. Samples are recovered by bringing down the entire trap system. Slowly untying the diagonal running rope, carefully leaving the traps on the soil, one over the other. Samples are gradually taken from the collection heads (Fig. 3C,D) as traps are at reach, the lower trap collection heads first.
2. After all traps are checked and samples removed, ethanol is added to the collection heads, first to the higher traps.
3. It is necessary to level the trap before it goes up; this is done by moving the leveling rope left or right at the prusik knot. After leveling, secure the knot to the rope with silver tape.
4. The cascade running rope is the only contact of the traps with the tree branch. We prevent ants from reaching the traps using silver tape with the sticky side out. The silver tape should be renewed every two or three months.

### Main system support (vertical cascade system)

**Fig. 4:**
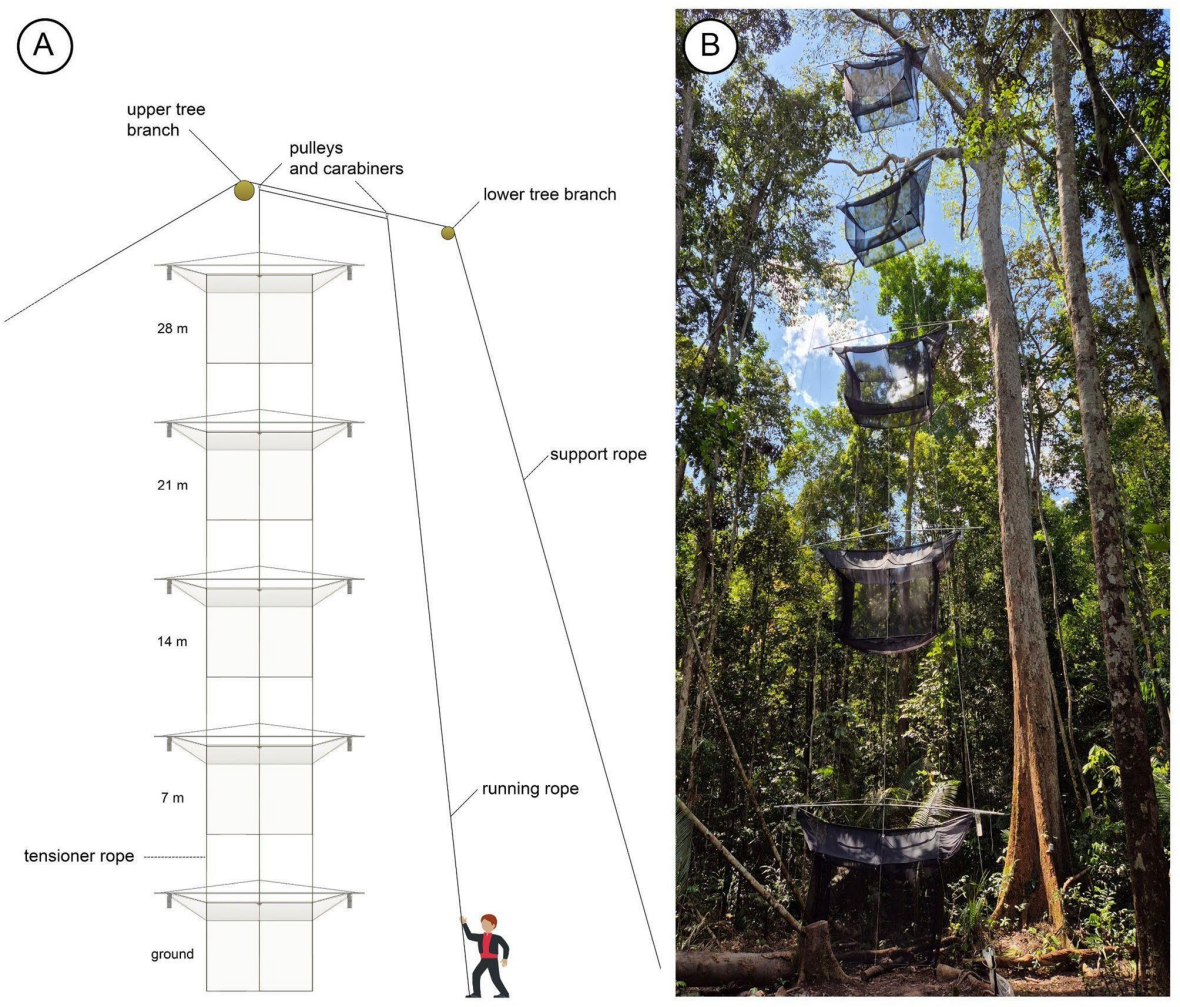
Vertical cascade system for interception flight trap. (**A**) Sketch of a complete vertical cascade system. (**B**) Complete vertical cascade system mounted at the ZF2 collecting point.

**Fig. 5:**
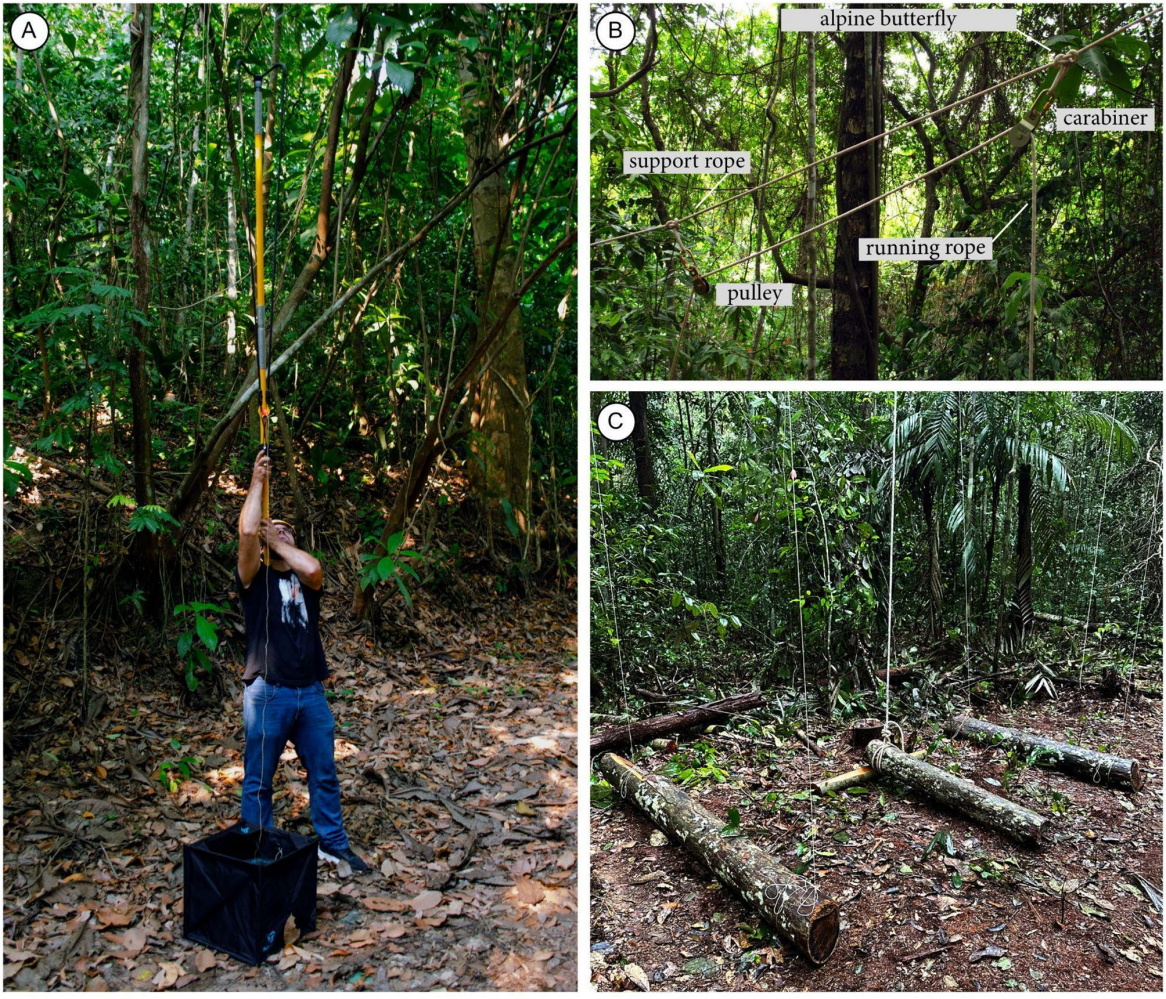
Fixing the modified Gressitt and Gressitt interception flight traps. (**A**) Folding cube and big shot with a trigger to loop a line over higher tree branches. (**B**) Assembly of the main structure of support ropes. (**C**) Fallen tree logs used as anchor points of the tensioner ropes (lateral) and running rope (central).

1. We use emergent trees (in the Amazon Basin, over 40–50 m high) to suspend and support the entire Cascade system (Fig. 4A, Supplementary Videos SV01, SV02 and SV03). The choice of tree species should consider how strong the large branches are. It is necessary to clean the ground below the cascade. With the overall weight, storms can bring down the entire system. A resistant branch over 30 m high (measured with a height telemeter) is necessary to install the higher trap so, in our project, its top is 28 m above the ground. Different techniques can be used to access the canopy^34^.
2. We use a big shot with a trigger (Fig. 5A) to throw the thin line (80 m long, 1.75 mm diameter, Dyneema fiber, lightweight, low stretch, high abrasion resistance, preferably in a high-visibility color) with a 220 g lead weight attached to one end, the other end inside a throw line folding cube (50 × 50 × 50 cm) (Fig. 5A). Shooting from close to the tree base allows choosing the best approach angle, which tends to minimize interference from branches and other trees. The lead weight may snap back at high speed, so a helmet should be worn.
3. After the line has been sent over the proper emergent tree branch, the cascade support rope replaces the throw line (80–100 m, 12 mm diameter, same model as the running rope). Many secure knots (clove hitch) tie the throw line to the support rope. A silver tape is passed at the connection point between the thin line and the stout rope, making the connection as narrow and smooth as possible to facilitate easy passage of these initial knots through narrow branch forks.
4. The position of the cascade support rope that will stay near the tree branch is flagged, and a first carabiner (steel carabiners 25KN with screw locks, 107 mm x 57 mm, or similar quality) is placed using an alpine butterfly knot; and the pulley (steel pulley with oscillating side plates to lift workload limit of 420 kg) is attached to the carabiner (Fig. 5B).
5. The second carabiner and respective pulley (Fig. 5B) (in the same way as described above) is fixed around 2 m from the first. The cascade support rope receives two pulleys placed more or less at half of the rope.
6. While on the floor, the running rope is passed through both pulleys and as they are hoisted, they carry the rope passed through them. Both ends of the running rope must be kept at the ground level to be managed while hoisting the trap structure. The pulley is lifted until the upper one reaches the tree branch.
7. A second shot should send a throw line to go over a second, lower, strong tree branch (about 25–30 m high), at least 10 m away from where the traps will be lifted, repeating step 2.
8. The throw line must be tied to the loose end of the cascade support rope, which is then pulled over the lower branch with the cascade support rope.
9. One pulley should be placed under (but close to) the upper tree branch, while the other stays diagonally slightly below, with the cascade running rope making a loop above, both ends long enough to reach the soil (Fig. 5B).
10. Pull up the cascade support rope to haul both pulleys up, with the cascade running rope passing in both. Stretch well and tie this rope to a safe anchor point (a stout trunk) close to the soil but distant from the vertical axis of the cascade. This way prevents the cascade support rope from touching the trap roof panels of the higher trap when the system is lifted.
11. Mark the point to fix the first trap in the cascade running rope. This trap will be higher than the other traps. Depending on the height of the tree branch and the number of traps to be installed vertically, the distance among the traps is calculated for all to be positioned equidistantly.
12. Fix the tensioner and running ropes in a safe anchor point at soil level (Fig. 5C) which will prevent the cascade system from moving laterally and will guarantee that the trap orientation stays along the east-west axis.

### Diagonal cascade system

**Fig. 6:**
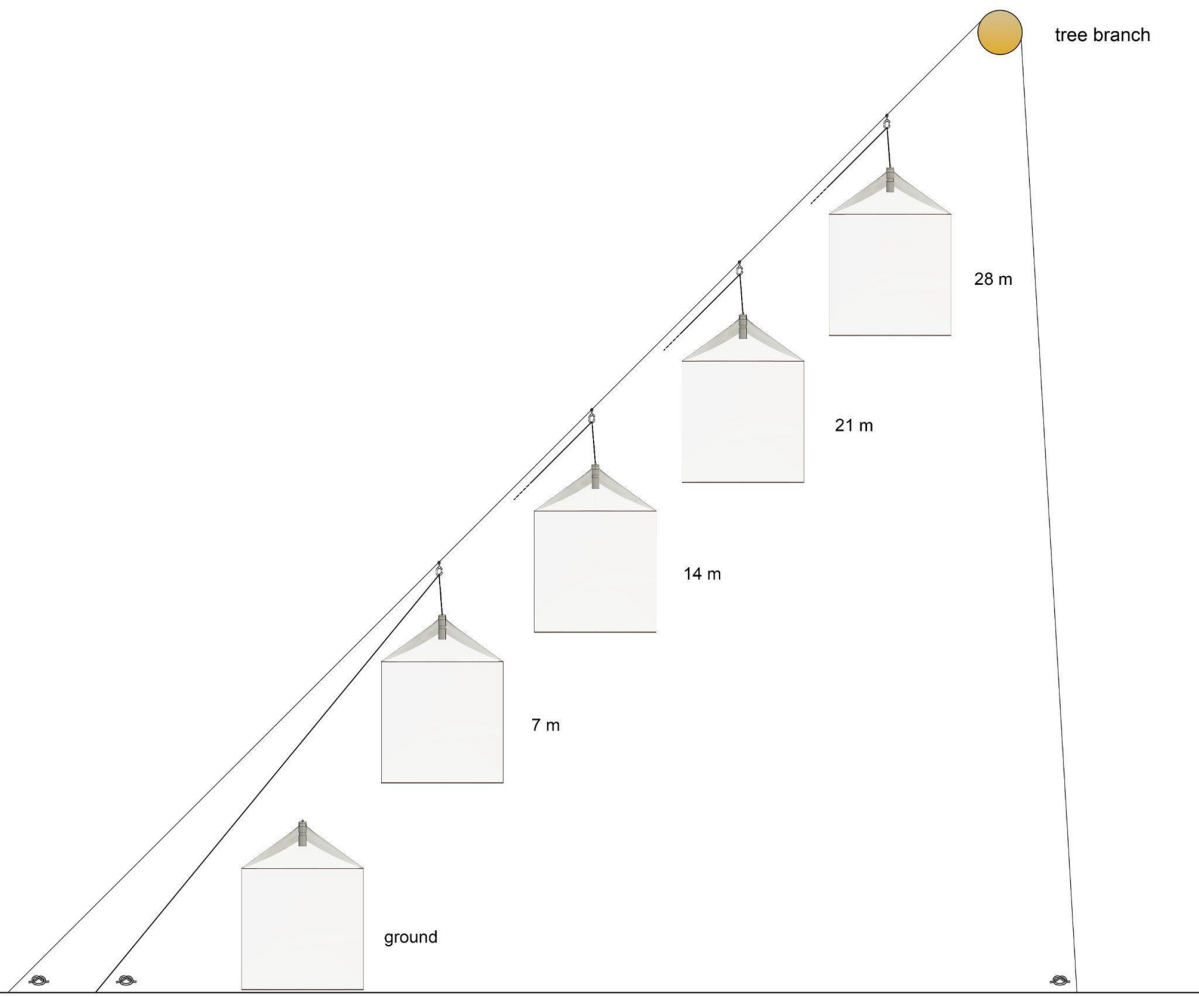
Sketch of the diagonal system of the modified Gressitt and Gressitt interception flight trap showing the traps at different heights and the support structures.

The cascade system using large interception traps can also be installed diagonally (Fig. 6) according to the conditions of the environment, such as canopy opening and tree height, as well as materials available. In the diagonal cascade system, the support rope attaches all pulleys. Each trap works independently and can be lifted and lowered with an independent cascade running rope. The traps do not need the roof and floor holes. The tensioner ropes will be tied in each trap and will work independently.

### Collecting points, emergent trees, and montage models

We presently have four areas explored using the cascade system, three verticalized near Manaus and one “diagonal system” in the Gurupi Biological Reserve, Maranhão. Point 1 is in the municipality of Manaus (about 50 km north of the city), on the ZF2 road, Cuieiras Biological Reserve (see Amorim et al.^15^. Point 2 is in the municipality of Iranduba, at the “Reserva de Desenvolvimento Sustentável Rio Negro”, about 100 km west of Manaus, west of the Rio Negro River. Point 3 is in Careiro Castanho (road to Autazes, km 11), about 50 km southeast of Manaus, south of the Amazon River. Point 4 operates in the state of Maranhão, in the Gurupi Biological Reserve, in a straight line about 1500 km east of Manaus. All four traps sample the vertical strata of the forest with traps at the ground level and the heights of 7 m, 14 m, 21 m, and 28 m above the ground.

In each of our collecting points, we found different emergent tree species used to support the trap system. In the sampling point 1, we used a large *angelim-pedra*—*Dinizia excelsa* Ducke (Fabaceae) (Fig. 4B), the largest tree in the Amazon. In the point 2, a tree of *arara-tucupi* or *faveira-grande*—*Parkia nitida* Miquel (Fabaceae). In the point 3, a tree of castanheira—*Bertholletia excelsa* Humboldt and Bonpland (Lecythidaceae). In point 4, a tree of apuí—*Ficus nymphaeifolia* Miller (Moraceae).

The vertical model (i) requires two people to perform maintenance since the traps are all interconnected by a single running rope, while in the diagonal model, the traps are independent. Therefore, maintenance can be performed by a single person, considering that the lowering and raising of the traps are independent. With the vertical model, forest removal is reduced, causing less environmental impact, while with the diagonal model, the environmental impact is about twice as high. To support the system, with the vertical model, approximately 100 to 200 m of 12 mm thick ropes (support and running ropes) are required, and approximately 140 m of thin ropes with approximately 0.2 mm thickness used to stabilize the system, while for the diagonal system, approximately 300 m of 12 mm thick rope are required to support the pulley system, the main one and those that support the four traps, and approximately 400 m of thin rope with 0.2 mm thickness used to stabilize the traps.

To standardize the labels of the samples, we recommend using different trap heights such as Cascade Ground, Cascade 7 m, and so on. This terminology aims to facilitate future discussions about possible flight patterns, with specimens collected using this methodology and later deposited in zoological collections.

The number of traps to be used in each collection point is a decision of each group, and what is important in this cascade system is to align vertically or diagonally the traps in one point to obtain the maximum biological information for each stratum of the forest. This trap setup depends on the location and needs a suitable tree for support. One tall tree, preferentially emergent, is enough to support all the traps.

## Supplementary Information

Below is the link to the electronic supplementary material.

Supplementary Material 1

Supplementary Material 2

Supplementary Material 3

Supplementary Material 4

## Data Availability

All data generated or analyzed during this study are included in this published article [and its supplementary information files].
