# Supplementary material for: Cascade of flight interception traps for large scale exploration of the otherwise unreachable canopy insect fauna
Source: Sci Rep. 2025 Oct 15;15:36029. doi: 10.1038/s41598-025-19981-w (PMC12528738; doi:10.1038/s41598-025-19981-w)
Supplement: Supplementary file 4 — Supplementary Material 4 [file 41598_2025_19981_MOESM4_ESM.docx]

**Legends**

Supplementary Video 1 – Time lapse of the cascade of traps installation.

Supplementary Video 2 – Downward view of the cascade of traps in the ZF2 area, north of Manaus, Amazonas, Brazil.

Supplementary Video 3 - Upward view of the cascade of traps in the ZF2 area, north of Manaus, Amazonas, Brazil.
